# Passing Events and Topics

**Published:** 1922-02

**Authors:** 


### PASSING EVENTS AND TOPICS.

IT is not easy to find anything new to say about the common—much too common—cold, but this feat has been accomplished by the "Daily Sketch's" tame humorist, "The Man in the Street." There is much to be said for his idea that we ought to have a new name for this infliction—preferably something that would suggest its connection with the fugging and the frowsting that so often produce it. "If colds had been called 'stuffiness' or 'bad airs,'" he says, "we would by now rarely have them, but that unfortunate name gives perpetual new life to the fallacy that a 'cold' is somehow connected with fresh air and a low temperature."

---

ACCORDING to Sir Arthur Keith, the square-jawed type which we like to think characteristically British is rapidly becoming rare. We are now a soft-food race, whose under-exercised jaws are relatively small and feeble. We will not quote Professor Keith's references to the already perceptible decline of female beauty—the subject is too painful. With greater calm, since we shall not survive to see it, we can contemplate the kind of face which the lady novelist of the year Something-in-five-figures, by which time all needless features will have disappeared, will give to the hero of her best-seller. Mr. H. G. Wells described it for us in an essay published as long ago as 1897 :—"Eyes large, lustrous, beautiful, soulful ; above them, no longer separated by rugged brow ridges, is the top of the head, a glistening, hairless dome, terete and beautiful ; no craggy nose rises to disturb by its unmeaning shadows the symmetry of that calm face, no vestigial ears project ; the mouth is a small, perfectly round aperture, toothless and gumless, jawless, unanimal, no futile emotions disturbing its roundness as it lies, like the harvest moon or the evening star, in the wide firmament of face."

---

THE first hospital in Birmingham to show a balance in hand is the seven-year-old institution known as the Birmingham and Midland Hospital for Nervous Disease. In the prevailing financial distress of hospitals in that city this would be by itself remarkable, but it is the more so because this hospital has been frowned upon from the start, and is alleged to have had no sufficient reason for existence. In reply, it claims to be the only large special hospital of its kind outside London. Last year it received 3,104 new patients, an increase of 500 on the previous year's total. The Lord Mayor lately declared that it was doing new work and relieving distress of a kind hitherto locally unprovided for. But the arguments for its existence, are success and solvency, an unusual combination at the present time. These deserve recording, and Mr. Joseph Ansell, the chairman, may be advised to make the most of the hospital conferences held in the city to adjust any surviving differences. It is often forgotten that co-ordination, which everyone preaches, and for which so few will make any sacrifice, may include additions to existing institutions. At all events co-ordination is much needed in a case like this.

THE Metropolitan District Committee of the National Federation of General Workers has issued a notice advising "all members of its affiliated unions not to subscribe to or help any demonstration on behalf of hospitals till they have agreed to pay trade union conditions to their staffs." This is the result of certain of the hospitals in London giving notice of the termination at the end of last year of an Award given by the Industrial Court in July, 1920. In other industries the wages of workers have been reduced, following on the decreased cost of living, and these hospitals in their action are merely following the lead of other employers of labour. If members of trade unions refuse their subscriptions and help to the hospitals, and these institutions suffer financially and are compelled to close their beds in consequence, then surely the trade unionist who is refused admission, in his need, to the hospitals, will only have himself and his affiliated unionists to blame. In the "Daily Herald" of January 10, there appeared the following headlines :—"No Hospital Help for the Dying.—Thousands Killed by Lack of Funds.—Pathetic Stories.—Tragedy of Ever-closed Doors." Well, a remedy for this is not to be found by the unions withholding their help and subscriptions !

---

THE Royal Devon and Exeter Hospital has recently been admonished in the columns of the Exeter paper, the "Express and Echo," as follows :—

> "The Hospital is maintained by the public to render medical and surgical aid to poor persons. There are many poor children in this City requiring treatment for adenoids. The Ministry of Health communicate with the Education Committee of Exeter and say : 'All those children ought to be treated, but it isn't fair to ask the Hospital funds to continue to bear the cost of those whose parents are unable to pay. We suggest that you arrange a fee ; we will pay half, your rate-payers must pay the other half.' The British Medical Association thereupon, possibly in consultation with the Ministry of Health, state what they consider to be an adequate fee, and the Education Committee offer to pay that fee. The Hospital Authorities at first agree to accept £1 1s. per child, where formerly they received nothing, but realising, after a few months, that the money was coming out of local rates and imperial taxes, they make a demand for £3 3s. per child ! Why ? Simply because there is the public pocket to dip into. If there is another explanation, I have not seen or heard it. Now, those who maintain the Hospital are also rate-payers and tax-payers, and there is some justifiable irritation that whilst they gave freely with one hand for the support of a charitable institution, they are expected to pay an unreasonably high fee for service when they, in their corporate capacity as rate-payers, approach that charitable institution with a mutual benefit proposition."

The circumstances, as quoted by our contemporary, show a lamentable state of affairs. This lack of co-ordination between the two public bodies, the Education Committee and the Hospital, can only be of dis-service both to the children who are waiting for operations and to the hospital which is dependent on the goodwill of the public. It is a matter for regret that no machinery exists for the adjustment of difficulties of this nature ; and we would suggest in this instance that both the contending parties should put their cases before an agreed referee and should abide by his decision.

**PASSING EVENTS AND TOPICS—(*cont.*).**

OUR racial humility—or perhaps a lack of that virtue in other nations—has induced our general belief that telephone tyranny is peculiar to Great Britain. It is quite refreshing to learn that the doctrine of the infallibility of telephone authorities is firmly held (by the authorities) in Paris. A well-known medical man's practice suffered severely from the fact that the number ascribed to him in the Telephone Directory really belonged to the Chamber of Deputies. So many would-be patients were baffled in the attempt to ring him up that he sued the State for damages, but, as anyone but an unreasoning optimist might have foreseen, it was held that the State in publishing the Directory entered into no contract to be accurate.

MOST people who are in a position to observe the attitude of the less-instructed portions of the community to infectious disease could match the story of the young woman recently fined at Bristol with tales of ignorance and selfishness almost as gross. This girl was suffering from scarlet fever. As there was no accommodation in the local fever hospital, she was permitted to stay at home, the doctor instructing her to remain in isolation for six weeks. Instead of doing this, she " went about the town, served in a shop, visited her fiancé, and travelled in a motor omnibus." It is hardly credible that no one knew what she was doing. The timidity of the uneducated in denouncing neighbours guilty of anti-social conduct is one of the reasons why proceedings of this kind can go on unchecked until all the mischief possible is done. And so it will be until a generation has grown up which has sufficient intelligence to believe, if not to understand, the teachings of science, and sufficient self-control and public spirit to live in accordance with its faith.

THE Minister of Health has appointed a Committee consisting of Sir Cyril Cobb, K.B.E., M.V.O. (chairman); Dr. R. P. Smith, M.D., F.R.C.P.; and Dr. Bedford Pierce, M.D., F.R.C.P., with Mr. P. Barter, of the Ministry of Health, as Secretary: " To investigate and report on the charges made by Dr. Lomax in his book (*The Experiences of an Asylum Doctor*) and to make recommendations as to any medical or administrative improvements which may be necessary and practicable in respect of the matters referred to by Dr. Lomax without amendment of the existing Lunacy Laws." The Committee will ordinarily hear evidence in public, and the time and place of meetings for this purpose will be announced in the Press; the Committee will, however, reserve the right to hear evidence in private in any case where they consider such a course desirable. The Committee will hear such evidence as is necessary for the investigation specified in the terms of reference; and cannot undertake to hear evidence in regard to the amendment of the existing Lunacy Laws.

ON another page we refer to the presentation of a purse to an institutional secretary in recognition of a long period of service, and argue the need for an adequate pension scheme for such officials. Another case in point is that of the family of the late Mr. Fletcher, financial secretary of the Royal Victoria Infirmary, Newcastle. A proposal that the infirmary should head the list of subscriptions on behalf of his widow was discarded, and a general subscription was decided. This is understandable, but if a pension scheme were arranged at the time of each officer's engagement, provision would be made in the ordinary course without raising questions of principle or exciting discussion. While an institution heavily in debt is bound to think twice before subscribing to private testimonials, at the same time it is hard that the families of its deceased officials should have to rely upon charitable appeals for their support when the breadwinner is taken from them. The proper principle here is the principle of pensions; and co-ordination is at least as necessary here as in any other department of hospital activity.

AN interesting article recently appeared in the Journal of the American Medical Association on the subject of spirochætosis of the respiratory tract. The authors came to the following conclusions: (1) Bronchial spirochætosis exists in the United States, and is probably more widespread than is generally recognised; (2) the disease in its clinical aspects is very suggestive of pulmonary tuberculosis, but usually can be readily distinguished from this infection; (3) the routine examination of all sputum should include a search for spirochætes, and their presence should be regarded as significant, unless proved otherwise; (4) the disease appears to be capable of transmission from an infected to a non-infected individual, although the degree of contagiousness is probably slight; (5) an individual harbouring the spirochætes in the sputum may present little or no evidence of the disease himself, and it appears that there exist carriers of *S. bronchialis*; (6) the disease, as a rule, responds readily to treatment, and the arsenical preparations, particularly arsphenamin or neoarsphenamin, are very efficacious. Pulmonary spirochætosis was recognised as occurring among our troops during the European War, and we cannot, therefore, suppose that the United States have a monopoly of this condition.

WE are glad to see that the Wingrove Hospital, under the control of the Newcastle Board of Guardians, has now been placed under separate administration from the workhouse. To work the change a new entrance has been made and formally opened by Mr. Walter Dix, chairman of the Board. Wingrove Hospital boasts of being the largest poor-law hospital between Leeds and Edinburgh. The work now demands a matron and three assistant matrons, and the hospital ward is for paying patients, who can be attended by their own practitioner. It has already, we understand, relieved the pressure upon the Newcastle Royal Infirmary; and the wonder is that this change is not universal in all poor-law hospitals of its size. Only by such separation can poor-law hospitals further their possibilities, and the most be made of our hospital resources whether voluntary or municipal.

